# Autophagy regulation using luteolin: new insight into its anti-tumor activity

**DOI:** 10.1186/s12935-020-01634-9

**Published:** 2020-11-04

**Authors:** Milad Ashrafizadeh, Zahra Ahmadi, Tahereh Farkhondeh, Saeed Samarghandian

**Affiliations:** 1 Faculty of Engineering and Natural Sciences, Sabanci University, Orta Mahalle, Üniversite Caddesi No. 27, 34956 Orhanlı, Tuzla, Istanbul, Turkey; 2 Sabanci University Nanotechnology Research and Application Center (SUNUM), 34956 Tuzla, Istanbul, Turkey; 3 Department of Basic Science, Shoushtar Branch, Islamic Azad University, Shoushtar, Iran; 4 Medical Toxicology and Drug Abuse Research Center (MTDRC), Birjand University of Medical Sciences (BUMS), Birjand, Iran; 5 Faculty of Pharmacy, Birjand University of Medical Sciences, Birjand, Iran; 6 Noncommunicable Diseases Research Center, Neyshabur University of Medical Sciences, Neyshabur, Iran

**Keywords:** Anti-tumor, Autophagy, Cancer therapy, Herbal medicine, Luteolin

## Abstract

Application of novel methods in cancer therapy is important in terms of management and treatment of the life-threatening disorder. It appears that autophagy is a potential target in cancer therapy, as a variety of drugs targeting autophagy have shown great potential in reducing the viability and proliferation of cancer cells. Autophagy is primarily a catabolic process which provides energy during starvation. Besides, this process contributes to the degradation of aged or potentially toxic components and organelles. On the other hand, the source of a variety of naturally occurring anti-tumor drugs are flavonoids which have high anti-tumor activity. Luteolin is a polyphenolic flavone with the great pharmacological effects such as anti-diabetic, hepatoprotective, antioxidant, anti-inflammation, and anti-tumor. At the present review, we demonstrate how luteolin affects on autophagy process to induce anti-tumor activity.

## Introduction

**Fig. 1:**
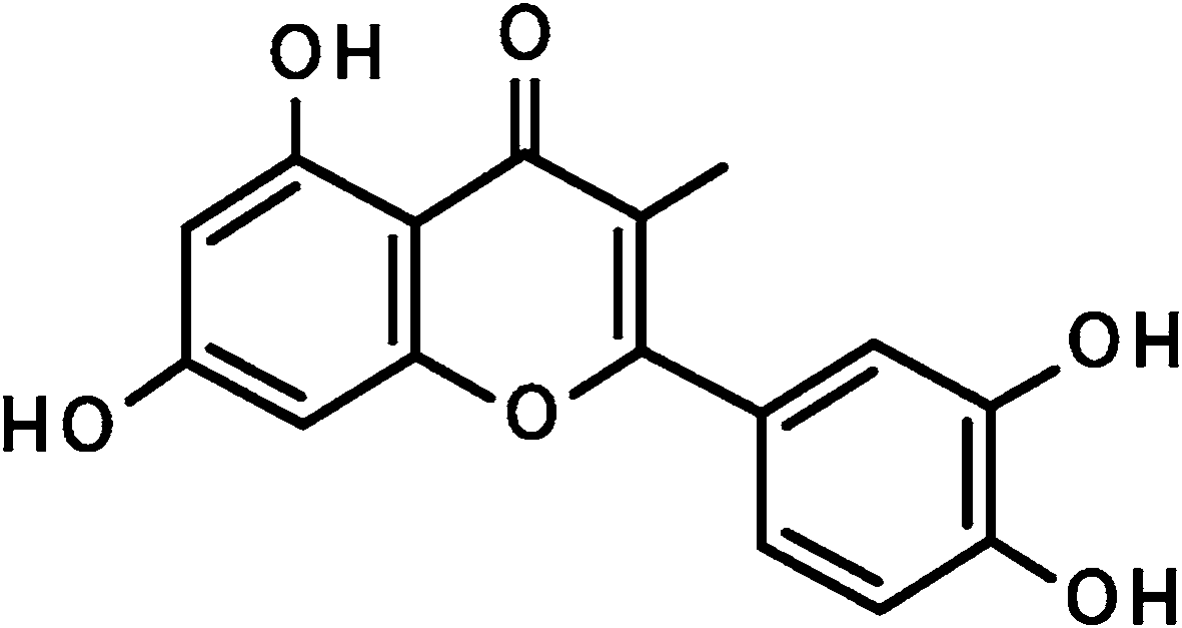
Chemical structure of luteolin

Cancer is defined as a condition that cells undergo unusual growth and obtain an invasive character. Although cancer has a long history, it appears that its incidence rate has enhanced in recent years. A variety of factors is involved in the induction of cancer and its progression. It has been demonstrated that lifestyle, exposure to potentially toxic agents, smoking, drinking, and lack of exercise are the potential factors causing cancer. So far, scientists have tried to provide synthetic drugs for management and treatment of cancer. However, it appears that synthetic anti-cancer drugs are not appropriate choices for cancer therapy. One of the most challenging factors is the high cost of these drugs, so that a lot of people around the world, particularly in developing countries, are not able to afford the cost of these drugs. On the other hand, the application of just a kind of drug for a long time reduces its effect and enhances the chance of resistance. Furthermore, some types of these drugs have high side effects, limiting their extensive application. Hence, scientists have focused on plant-derived chemicals due to their excellent pharmacological effects and minimal side effects [1–4]. Flavonoids are the secondary natural metabolites possessing a polyphenolic part and have great health-promoting effects [5]. Luteolin (3′, 4′, 5, 7-tetrahydroxyflavone) is a polyphenolic flavone existing in various types of herbs, vegetables, and fruits. Luteolin with chemical formula C15H10O6 is a tetrahydroxyflavone in which the four hydroxy groups are present at positions 3′, 4′ 5, and 7 (Fig. 1) [5]. Luteolin has an important place in traditional Chinese medicine[6]. This naturally occurring nutraceutical compound has wide potentials such as anti-inflammatory, anti-apoptotic, antioxidant, and anti-cancer. Luteolin has an efficient neuroprotective capability and is able to ameliorate brain trauma and spinal cord damage caused by 1-methyl-4-phenylpyridinium (MPP) [6]. The epithelial-to-mesenchymal transition (EMT) is inverted by luteolin via contracting of the cytoskeleton, increasing the expression of biomarker E-cadherin as well as decreasing the expression of biomarkers snail, vimentin, and N-cadherin. Reactive oxygen species (ROS) in intracellular space of glioblastoma cells are accelerated by luteolin through the mechanisms consisting of endoplasmic reticulum [7] stress activation, the disabling of mitochondria, and increasing the expressions of ER stress-associated proteins such as ATF4, CHOP, PERK, eIF2α and cleaved-caspase 12 [8]. The capability of luteolin in affecting molecular signaling pathways has been investigated and it seems that this plant-derived chemical is able to influence the differentiation of osteoclasts, and myeloid and erythroid cells[9]. Besides, it has been found that luteolin can sustain the pluripotence of periodontal ligament cells by targeting the Oct4/Sox2 pathway[10]. Luteolin can hamper the activity of NADPH oxidase (NOX), decrease the level of lipid peroxidation, and decrease the depletion of glutathione and ROS generation. The regulation of these processes is performed in a dose-dependent manner [11]. Luteolin has shown nephroprotective activity by decreasing oxidative stress, the number of apoptotic cells, and the level of nephron damage [12]. In terms of anti-tumor activity, luteolin diminished the phosphorylation of ERK and FAK signaling pathways to prevent the progression and proliferation of ovarian cancer cells [13]. There is a need for detecting new pathways in cancer therapy. Stimulation of apoptotic cell death is conventionally applied in cancer therapy. However, some studies have demonstrated that induction of apoptosis may enhance the proliferation of living cancer cells and it has been reported that some tumor cells are resistant to apoptosis [2, 3]. It seems that other ways are used in diminishing the viability and malignancy of cancer cells. Autophagy is one of the potential candidates in cancer therapy. Multiple studies have confirmed the efficiency of the autophagy mechanism in cancer therapy. Therefore, there have been some efforts to develop anti-tumor drugs targeting autophagy. In respect to the modulatory effects of luteolin on different signaling pathways, it appears that molecular signaling pathways of autophagy can be regulated by luteolin. Furthermore, based on the role of autophagy in different disorders and maintaining physiological conditions, the effect of luteolin on autophagy leads to therapeutic results. At the present review, we focused on the regulatory impact of luteolin on autophagy and demonstrate how luteolin affects autophagy to diminish the viability, proliferation, migration, and malignancy of cancer cells.

## Autophagy mechanismm

**Fig. 2:**
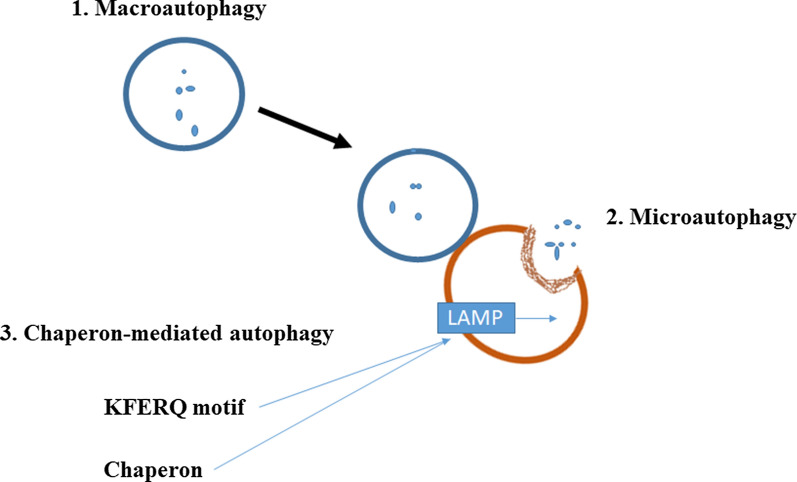
Categorized of autophagy process

Autophagy is a Greek word meaning “self-eating”. This phenomenon was first expressed by Christian de Duve around 40 years ago. Autophagy was seen during the destruction of mitochondria and other cytoplasmic organelles within lysosomes of rat hepatocytes [14]. Autophagy has an indispensable role in the homeostasis of the eukaryote cells during the stress conditions [15]. The Nobel Prize in 2016 was dedicated to a Japanese scientist for great work in detecting the cellular mechanism of autophagy. This mechanism is vital in the treatment of a variety of diseases [16]. Aged or damaged organelles and abnormal proteins are transmitted into lysosomes to undergo degradation. The obtained components or amino acids are recycled by the cell [17]. Autophagy process is categorized into three types, known as microautophagy, macroautophagy, and chaperone-mediated autophagy (CMA), (Fig. 2) [18]. In macroautophagy, the cargo is surrounded by cytosolic double-membrane vacuoles, “autophagosomes”, transmitting to the lysosome for decomposition [19]. Microautophagy is the non-selective lysosomal degradative procedure in the cell. During microautophagy, autophagosome is not formed and membrane invaginations surround cargo [20]. In CMA, the soluble chaperones selectively deliver proteins to the lysosome [21]. Apart from three classic types of autophagy, there are some selective particular forms containing pexophagy, mitophagy, xenophagy, ribophagy, and secretory autophagy [22].

## Autophagy and relevant signaling pathways

**Fig. 3:**
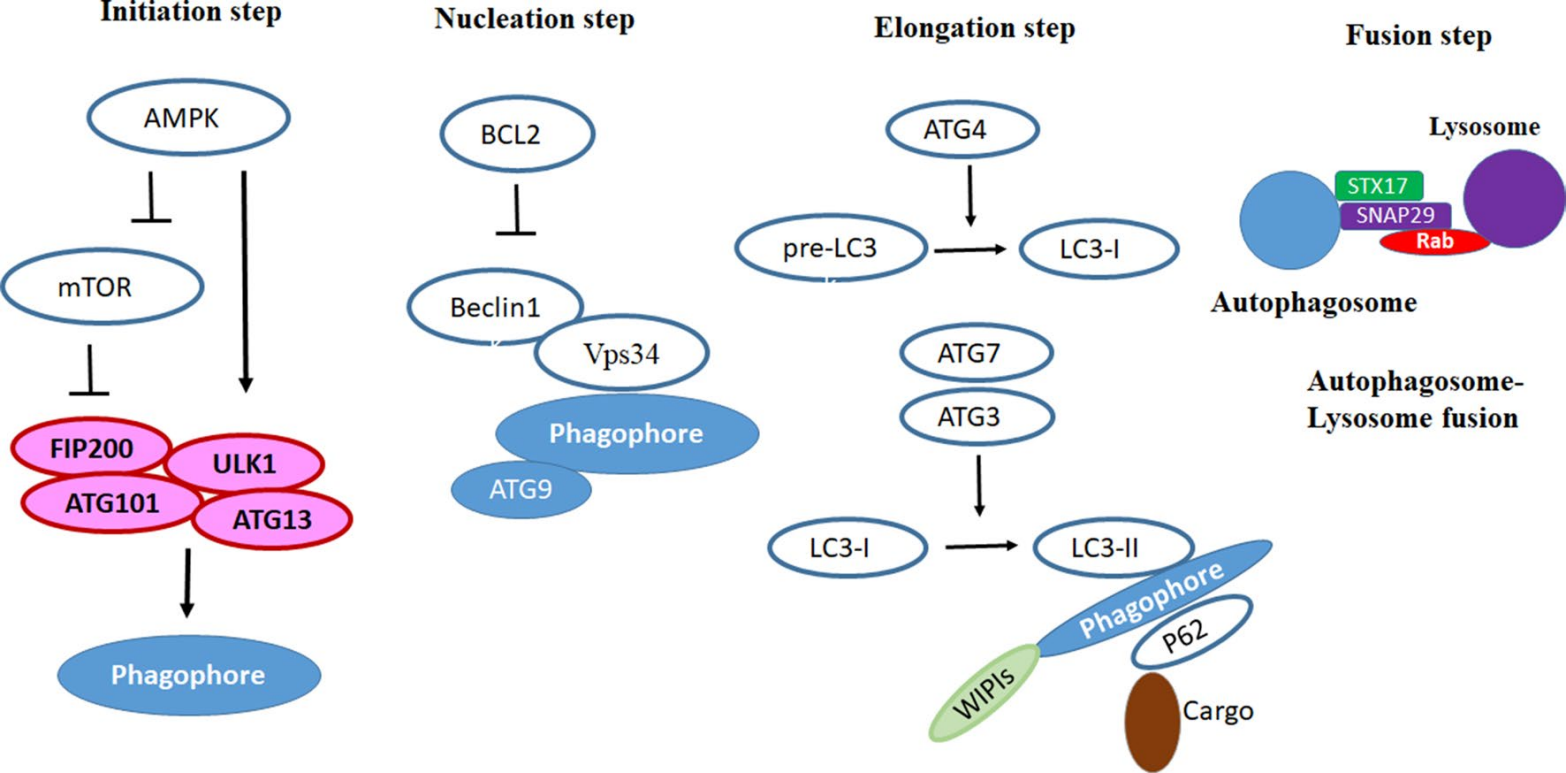
The molecular pathways of autophagy

The molecular pathways of autophagy have comprehensively been described in two excellent reviews by Kroemer and Galluzi [23, 24]. The autophagy mechanism is divided into four major steps: (A) initiation, (B) nucleation, (C) elongation, and (D) fusion. At the initiation step, a complex containing FIP200, autophagy-related gene (ATG)-13 and Unc-51 Like autophagy activating Kinase 1 (ULK1) is involved in the induction of phagophore formation. Besides, class III phosphatidylinositol 3-kinase complex I (PI3KC3-C1) triggers the formation of phagophore [25]. It seems that the mammalian target of rapamycin (mTOR) functions as the upstream mediator of FIP200/ATG13/ULK1 complex. At the energy sufficiency, the mTOR pathway effectively suppresses this complex. However, nutrition deprivation leads to the inhibition of mTOR, resulting in activation of FIP200/ATG13/ULK1 complex and induction of phagophore formation. AMP-activated protein kinase (AMPK) is able to modulate the mTOR pathway. AMPK acts as a sensor of AMP and an increase in the ratio of AMP/ATP activates AMPK. As a consequence, AMPK inhibits mTOR, resulting in FIP200/ATG13/ULK1 activation and phagophore formation. In the nucleation step, Beclin1 and vacuolar protein sorting-34 (Vps34) involve in this step. At the third step (elongation), ATG4 produces LC3-I from pre-LC3, and next, ATG7 and ATG3 result in the formation of LC3-II from LC3-I. There are several lines of evidence that ATG12 has a significant role in the elongation step [23]. Finally, at the fusion step, Syntax 17 (STX17), Synaptosome associated protein 29 (SNAP29), vesicle-associated membrane protein 8 (VAMP8), and Rab7 stimulate the fusion of lysosome and autophagosome (Fig. 3), [24].

## Autophagy and disease development

Dysfunction in the autophagy process is related to different types of diseases like muscular disorders (Danon’s disease), cerebral ischemia, neurodegeneration, pathogen infections (Listeria monocytogenes), and cancer. Disturbance of autophagy leads to psychotic symptoms, mood disorders, and behavioral change [26]. Activation of autophagy is associated with restriction of tumorigenesis and prevention of damage in genome cells [27]. Recently published scientific research shows that breast and ovarian cancers are associated with Beclin1 gene mutation [28]. In Parkinson’s disease (PD), mitophagy is declined by the PINK1 mutation [29]. The activity of autophagy is mutated in the pancreatic β cells in diabetes mellitus and insulin resistance [30]. Autophagy is a substantially important mechanism occurring in the endothelial cells of blood vessels during nutrient deprivation, hypoxic, and pro-angiogenic tumor conditions [31]. Autophagy regulation impresses the melanocytes, skin fibroblasts, and keratinocytes in the conditions of skin diseases, therefore, it can be applied in therapeutic methods [32]. The discussion about the potential role of autophagy in pathological conditions is out of the scope of this article and a great review by Kroemer and colleagues has been conducted to examine the involvement of autophagy in different disorders [24].

## Regulation of autophagy by luteolin in cancer cells

Luteolin could affect various signaling molecules involved in autophagy process including nucleation and elongation and prevent cancer progression. Here, we discussed the available studies on the effect of luteolin on autophagy regulation in cancer models.

### Luteolin on nucleation and elongation steps in autophagy

Beclin1 is the main regulator of autophagy specifically in nucleation step, leading to tumor suppression [33]. Luteolin (100 µM) could down-regulated beclin1 expression in PC12 cells (derived from a pheochromocytoma of the rat adrenal medulla), leading to inhibition of autophagy. Luteolin could affect the expression of the endoplasmic reticulum (ER) chaperone binding immunoglobulin protein, activating ER stress sensors (eukaryotic initiation factor 2α phosphorylation and Xbox-binding protein 1 mRNA splicing) and inhibited autophagy [34]. Beclin1 silence promoted microtubule-associated protein 1 light chain 3-II (LC3-II) protein formation and increased punctate fluorescent signals in Miapaca2 cells transfected with a green fluorescent protein (GFP)-tagged LC3. In the study conducted by Kwon et al. (2017) on PC12, they found also luteolin (100 µM) was able to stimulatory effect on autophagy in elongation step through downregulation of LC3 [34].

Silibinin (50 µM) plus luteolin (20 µM) inhibited the growth of glioblastoma cells. The co-administration of silibinin and luteolin completely inhibited invasion and migration. In addition, silibinin (50 µM) plus luteolin (20 µM) inhibited rapamycin (RAPA)-induced autophagy via PKCa suppression and also apoptosis through an increase in the miR-7-1-3p expression in glioblastoma cells. Animal studies approved that overexpression of tumor suppressor, miR-7-1-3p, increased anti-tumor activities of silibinin (50 µM) and luteolin (20 µM) in RAPA pre-treated both U87MG and T98G tumors. Thus, these results clearly showed that miR-7-1-3p overexpression increased the anti-tumor activities of silibinin (50 µM) and luteolin (20 µM) to inhibit autophagy and induce apoptosis for controlling the growth of different human glioblastomas in vivo. Thus, autophagy can also act as a cell-survival mechanism in glioblastoma cells via Beclin-1 down-regulation [35].

Luteolin also regulates autophagy involved in the sensitization of cancer cells to apoptosis [35]. Luteolin (20 µM) induce sensitization of human liver cancer cells to tumor necrosis factor-related apoptosis-inducing ligand (TRAIL)-induced apoptosis through autophagy by decreasing the level of p62 and up-regulation of JNK-mediated death receptor. Chloroquine suppressed autophagy stimulated by luteolin. It was suggested that inhibition of autophagy prevented the anti-tumor activity of luteolin [36]. Therefore, autophagy may act as an anti-tumor mechanism and such a strategy is applied by luteolin in decreasing the viability and proliferation of human multiple myeloma cells [36]. It has also found that luteolin markedly inhibited RPMI-8226 cell proliferation dose-dependently. Luteolin (40–80 µmol/L) for 24 h and 20–80 µmol/L for 48 h could inhibit proliferation in a myeloma cell line (RPMI-8226 cell). Luteolin LC3-II in (20 µmol/L for 48 h) significantly elevated the expression of cleaved caspase3 and Ln RPMI 8826 cells exposed to the chloroquine concurrent. Luteolin inhibited the proliferation in the RPMI-8226 cells through stimulating apoptosis and autophagy [36]. As an example of cell-survival activity, ovarian cancer cells stimulate autophagy to induce resistance against cisplatin treatment [37]. As a consequence of cisplatin administration, the viability of ovarian cancer cells was improved through putative serine/threonine protein phosphatase (PRPA1)-medicated autophagy. Luteolin enhanced cisplatin-mediated apoptosis in a dose- and time-dependent manner. Mechanistically, this reduction in viability and proliferation was accompanied by autophagy inhibition via reducing LC3-II levels. Luteolin suppressed autophagy by a decrease in the expression of LC3-II, but decreased the cell death induced by cisplatin, resulted in the increase of the sensitivity of ovarian cancer cells to cisplatin. Indeed, cisplatin increased cell survival by increasing PARP1. PARP1 siRNA increased luteolin -induced inhibition of cell vitality and the sensitivity to cisplatin with reduced LC3-II levels. It has been shown that luteolin can suppress autophagy but enhance by decreasing apoptosis in ovarian cancer [37].

Luteolin decreased proliferation via inducing MAPK activation, leading to apoptosis and autophagy in the glioma cells in a time- and dose-dependent manner. Luteolin induced the death receptor (FADD) to modulate the apoptosis proteins including caspase-8, -3, and PARP. Luteolin elevated the expression levels of LC3B II/I and down-regulated the level of p62, leading to autophagy. qRT-PCR approved that luteolin upregulated the expression of miR-124-3p [37].

A study conducted by Cao and colleagues on the hepatocellular carcinoma (HCC) revealed a new pathway of luteolin action [38]. As one of the most common tumors of liver, HCC therapy is in priority. Induction of apoptotic cell death is a well-known way for reducing the viability and proliferation of HCC. Luteolin is able to significantly reduce the malignancy of HCC cells. It appears that luteolin administration elevates apoptosis in HCC cells by upregulating the expression of caspase 8 and downregulating the expression of BCL-2, an anti-apoptotic factor. Investigating the molecular pathways revealed that luteolin exerts a stimulatory impact on autophagy by enhancing the expression of Beclin-1. Inhibition of autophagy using chloroquine abrogated the impact of luteolin on cell apoptosis. Luteolin applies autophagy as a complementary process to sensitize HCC cells into apoptotic cell death [38].

Luteolin (25, 50, or 100 µM) stimulated apoptosis in human liver cancer (SMMC-7721 cells), especially via autophagy. Therefore, luteolin could regulate autophagy in HCC. Luteolin decreased the viability of SMMC-7721 cells, time and dose-dependently, and caused an arrest in G0/G1-phase. Additionally, flow cytometry analysis and Hoechst 33,342 staining indicated that luteolin elevated the number of apoptotic cells, and qRT-PCR and western blotting indicated that luteolin elevated caspase 8 and reduced BCL-2 at the mRNA and protein levels. In addition, luteolin elevated the number of autophagosomes, induced LC3B-I conversion to LC3B-II, and elevated the expression of Beclin 1. Co-administration of chloroquine, autophagy inhibitor, decreased the effects of luteolin on cell apoptosis [38].

Chemotherapeutic agents such as doxorubicin (DOX) are extensively applied in the treatment of osteosarcoma. It has been reported that autophagy stimulation is exerted by DOX in osteosarcoma cells. Luteolin can be used as an adjuvant in osteosarcoma therapy [39] Luteolin (100–200 µM) potentiated autophagy induced by doxorubicin in human osteosarcoma U2OS cells via up-regulating beclin1. The concurrent administration of luteolin and DOX changed cytoplasmic LC3-I to the membrane-bound form (LC3-II). Thus, autophagy induction is enhanced by co-administration of DOX and luteolin in human osteosarcoma (U2OS cells) [39].

Luteolin (5 and 10 mmol/L) increased the viability of p53- null Hep3B cells by inducing autophagy. Luteolin up-regulated the protein level of LC3-II and down-regulated p62 w in p53 null Hep3B cells. Thus, it has been found that luteolin-induced autophagy, leading to stimulation of cancer cell survival. It was found that luteolin-induced autophagy inhibited a decrease of cell number by co-administration of autophagy inhibitors [38].

Luteolin (50 mmol/L) caused cytoprotective autophagy in cutaneous squamous cell carcinoma cells. Ultrastructural analysis by transmission electron microscopy (TEM) indicated that luteolin elevated presence of autophagosomes filled with cargo in MET4 cells treated with luteolin. An elevation in autophagosome deposition may lead to the autophagosome formation or inhibition of autophagic degradation. The GFP-tag will be quenched rapidly in the acidic environment of the autolysosome, leaving only the mRFP-tag detectable. The deposition of mRFP punctae in the absence of green fluorescence in luteolin-treated MET4 cells, showing an elevated autolysosome formation and increased autophagic flux. Autophagic flux was assessed by reducing p62 levels. Luteolin at higher concentration (> 50) reduced p62 levels in MET4 cells, indicating that luteolin elevated the autophagic flux. Co-administration of chloroquine blocked lysosomal degradation and preserved LC3-II and p62. Thus, luteolin caused autophagy in the MET4 cell. Blockage of the late phase of autophagy increased the susceptibility of MET4 cells to luteolin-induced apoptosis. Co-administration of chloroquine and luteolin increased apoptosis and potentiated caspase-3 and Parp cleavage [40].

Luteolin at high concentration (200 mmol/L) caused an induction in lethal autophagy in lung carcinoma cells. Luteolin showed the anti-cancer activity in human lung carcinoma cells (NCI-H460) through inducing caspase-dependant apoptosis. The α subunit of the eukaryotic initiation factor 2 (eIF2α/C/EBP homologous protein pathway) has the main role in induction of apoptosis by the administration of luteolin. Treatment with luteolin also induces autophagy by the elevation of LC3 puncta and also enhanced autophagy flux. Additionally, blocking autophagy by bafilomycin A1 decreased apoptotic cell death, indicating that luteolin-induced autophagy by activation of caspases and independent Beclin-1 [41].

Luteolin disturbed hypoxia adaptation in colon cancer cells (HCT116) and in the human breast adenocarcinoma cell line (MDA-MB231), by inducing apoptosis and autophagy; however, autophagy was a part of protective effect, rather than involvement in the cytotoxic effect. Luteolin (25 and 50 µM) increased LC3-II both in the presence and absence of CoCl2, in HCT116 cells, suggesting that luteolin can cause an induction in apoptosis and autophagy in control and CoCl2-incubated HCT116 and MDA-MB231 cells. Bafilomycin A1 BaA1, an ATPase inhibitor, induced an autophagosome accumulation, regardless to the presence of CoCl2, suggesting that autophagic flux contributed to the luteolin effect [42].

Luteolin (1, 2, 5, 10 µM) decreased the viability of metastatic human colon cancer cells (SW620) and induced autophagy through increasing in the expression of beclin-1, autophagy-related protein 5 (Atg5), and microtubule-associated protein 1A/1B-light chain 3 beta-I/II (LC3B-I/II).

**Fig. 4:**
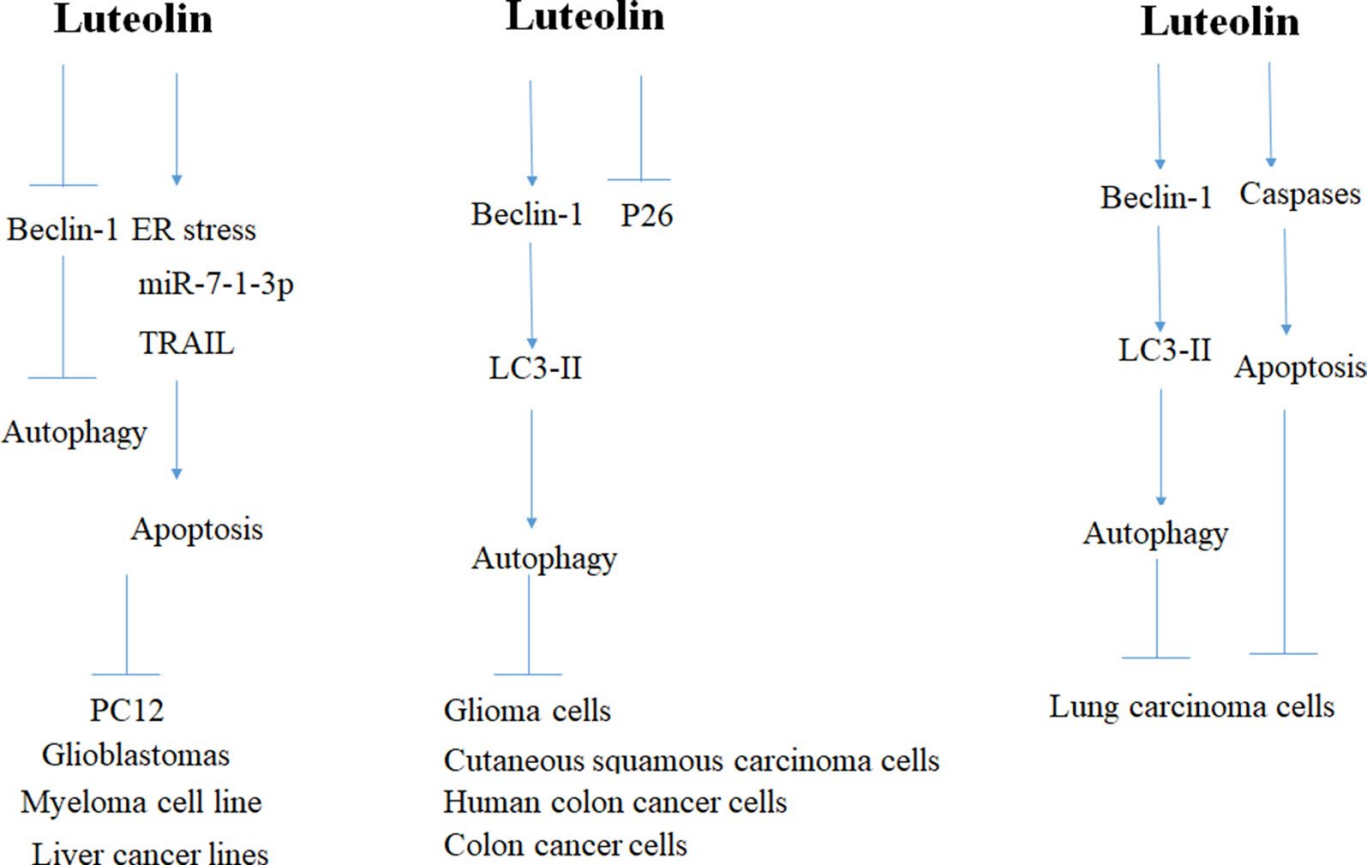
Regulation of autophagy by luteolin in cancer cell lines. Luteolin inhibited cancer cell lines progression through autophagy, apoptosis or both pathway

Luteolin induced reversal of the epithelial-mesenchymal transition process via the inhibition of the wingless-related integration site protein (Wnt)/β-catenin. The cytotoxic effect of luteolin was accompanied with extracellular signal-regulated kinase 1/2 (ERK1/2) and forkhead box O3a (FOXO3a) activation. Administration of the mitogen-activated protein kinase kinase (MEK) inhibitor PD0325901 blocked ERK-dependent FOXO3a phosphorylation, leading to an increase in FOXO3a expression and apoptosis, and a decrease in autophagy activity. The antitumor effect of luteolin in SW620 cells was related to the ERK/FOXO3a [43] (Fig. 4; Table 1).

**Table 1:** Modulatory effect of luteolin on autophagy in cancer cell lines

Drug	Cancer cell line	IC50	Effect on autophagy	Dose	Period of experiment (h)	Results	Ref
Luteolin	PC-12	–	Inhibition	100 µM	24	Reducing tumor proliferation	[34]
Luteolin	Liver cancer cells	–	Inhibition	20 µM	24	Sensitized human liver cancer cells to TRAIL-induced apoptosis	[35]
Luteolin	Human Multiple Myeloma Cell RPMI-8226	–	Induction	20–80 µmol/L	24 and 48	Providing a decrease in the proliferation of tumor cells	[36]
Luteolin and cisplatin	Ovarian cancer cell	–	Inhibition	20 and 40 µM	24	Promoted the sensitivity of cisplatin in ovarian cancer by decreasing PRPA1-medicated autophagy	[37]
Luteolin	SMMC-7721 cells	–	Induction	0, 12.5, 25, 50, 100 and 200 µM	24, 48 and 72	Improving apoptotic cell death	[38]
Luteolin and doxorubicin	Human osteosarcoma U2OS cells	–	Induction	10, 50, 100 and 200 µM	24	Improving doxorubicin-mediated autophagy and decreasing osteosarcoma viability	[39]
Luteolin and Chloroquine	Cutaneous squamous cell carcinoma (MET4 cell)	–	Induction	50 mmol/L	24	Chloroquine enhanced the cell death inducing effect of the flavonoid luteolin in metastatic squamous cell carcinoma cells	[40]
Luteolin	NCI-H460 lung carcinoma cells.	–	Induction	200 mmol/L	24	Induction of endoplasmic reticulum stress-mediated apoptosis and non-canonical autophagy	[41]
Luteolin	Colon cancer cells (HCT116)	27.61 ± 1.28	Induction	25 and 50 µM	24	Impaired hypoxia adaptation and progression in human colon cancer cells	[42]
Luteolin	Human colon cancer (SW620) cells	48.8 ± 1.6 µM	Induction	1, 2, 5, and 10 µM	24	Induction of forkhead box O3a (FOXO3a) activation-medited autophAGY	[43]

## Regulation of autophagy by luteolin in experimentally induced Solid Ehrlich Carcinoma

Luteolin plus 5-FU administration elevated p53, p21, caspase 3 and damage regulated autophagy modulator (DRAM) in Solid Ehrlich Carcinoma (SEC) model. It was also found that tumor weight and volume, the activity of thioredoxin reductase one (TR1) and cyclin D1 expression as well as oxidative indices were ameliorated [44]. An in vivo study on the protective effects of luteolin against U87MG and T98G xenografts in athymic nude mice indicated that luteolin (10 mg/kg/day), or silibinin (200 mg/kg/day), or their combination inhibited glioblastoma cells growth due to induction autophagy and activation of the caspases for induction of both the extrinsic and intrinsic pathways of apoptosis [45].

## Preventive effect of luteolin against cancer in human

Flavonoids may be effective against cancer progression via several mechanisms including autophagy modulation. A prospective study assessed the association between the intake of some flavonoids including luteolin and risk of cancers among 38,408 women above 45 years during 11.5 years of follow-up. They did not found the association between flavonols consumption and cancer prevention [46].

## Conclusion and remarks

Flavonoids such as luteolin are potent antioxidants and transient metals chelators that contributed in oxidation. Flavonoids are lipophilic agents that can interact with the cell membrane and penetrate into cell. Flavonoids have hydroxyl groups that cause some polarity and low acidic property for the molecule. The low pH of flavonoids helps them to penetrate into the lipid bilayer.

In addition, the complex of flavonoids with metals may act as molecular “fasteners”. These complexes have a role in the penetration into the hydrophobic sites of membranes and begin their adhesion. This pharmacological activity of flavonoids is important for their protective effects against cancer [47]. After penetration into cell, flavonoids can bind to actin in the cytoplasm and nucleus of cancerous cells and induce molecular control mechanisms against cancer [48].

It seems that the administration of luteolin in cancer therapy is advantageous and modulation of autophagy is one of the ways that luteolin follows to diminish the viability and proliferation of tumor cells. The findings have shown that luteolin dually down-regulates/upregulates autophagy in cancer therapy. So far, scientists have not found a pattern of effect of luteolin on autophagy. However, this study demonstrates that the impact of luteolin on autophagy depends on the action of autophagy in tumor cells. For instance, in a study earlier mentioned in this review [42], autophagy plays as a survival mechanism in glioblastoma cells, and in this case, autophagy exerts an inhibitory effect on autophagy. In contrast, if the autophagy decreases the viability and proliferation of tumor cells, luteolin positively affects autophagy to improve the reduction in malignancy of cancer cells as what happened in osteosarcoma cells [39]. Taken together, the concentration might be an important factor in determining whether luteolin-induced autophagy induces or inhibits cell death. However, more studies are needed to demonstrate the effect of luteolin on autophagy.

## Data Availability

Not applicable.

## Abbreviations

MPP: 1-Methyl-4-phenylpyridinium
EMT: Epithelial-to-mesenchymal transition
ROS: Reactive oxygen species
ER: Endoplasmic reticulum
NOX: NADPH oxidase
CMA: Chaperone-mediated autophagy
ATG: Autophagy-related gene
ULK1: Unc-51 like autophagy activating kinase 1
PI3KC3-C1: Class III phosphatidylinositol 3-kinase complex 1
mTOR: Mammalian target of rapamycin
AMPK: AMP-activated protein kinase
Vps34: Vacuolar protein sorting-34
STX17: Syntax17
SNAP29: Synaptosome associated protein 29
VAMP8: Vesicle-associated membrane protein 8
PD: Parkinson’s disease
PRPA1: Putative serine/threonine protein phosphatase 1
HCC: Hepatocellular carcinoma
DOX: Doxorubicin
miRNA: MicroRNA
